# Seasonal Dynamics and Pathogen Diversity of Tick Species Parasitizing Migratory Birds in Sardinia, Italy: Implications for the Spread of *Rickettsia*, *Babesia*, and *Theileria* Species

**DOI:** 10.3390/vetsci12080753

**Published:** 2025-08-13

**Authors:** Chisu Valentina, Laura Giua, Piera Bianco, Giovanna Chessa, Cipriano Foxi, Gaia Muroni, Giovanna Masala, Ivana Piredda

**Affiliations:** 1 Istituto Zooprofilattico Sperimentale della Sardegna “G. Pegreffi”, Via Duca degli Abruzzi 8, 07100 Sassari, Italy; laura.giua@izs-sardegna.it (L.G.); piera.bianco@izs-sardegna.it (P.B.); giovanna.chessa@izs-sardegna.it (G.C.); cipriano.foxi@izs-sardegna.it (C.F.); giovanna.masala@izs-sardegna.it (G.M.); ivana.piredda@izs-sardegna.it (I.P.); 2 Istituto Zooprofilattico Sperimentale della Sardegna “G. Pegreffi”—Centro di Sorveglianza Epidemiologica, Via XX Settembre 9, 09125 Cagliari, Italy; gaia.muroni@izs-sardegna.it

**Keywords:** migratory birds, *Ixodes ricinus*, *Rickettsia* spp., *Babesia*/*Theileria* spp., tick-borne zoonoses, One Health surveillance

## Abstract

Migratory birds play a key role in the ecology of tick-borne pathogens, serving as both hosts for ticks and as potential carriers of a wide range of infectious agents that can affect wildlife, domestic animals, and humans. Their long-distance movements contribute to the dispersal of ticks and the pathogens they harbor, with potential implications for the emergence and spread of zoonotic disease. This study focuses on the prevalence of *Rickettsia* spp. and *Babesia/Theileria* spp. in ticks collected from migratory birds in Sardinia, Italy, during two consecutive migration seasons (April–May and October–November 2021), corresponding to the spring and autumn migratory periods. A total of 961 ticks, primarily *Ixodes ricinus*, was collected from various bird species. Molecular analyses using polymerase chain reaction (PCR) and sequencing enabled the detection and identification of multiple *Rickettsia* species, with *R. helvetica*, *R. monacensis*, and *R. aeschlimannii* being the most frequently identified. Protozoan pathogens, including *B. venatorum* and *Theileria ovis*, were also detected in the tick samples. These findings underscore the diversity of pathogens in bird-associated ticks and the role of migratory birds in the geographical spread of these diseases. These results also provide valuable insights into pathogen transmission dynamics and stress the importance of monitoring migratory birds to assess and mitigate the risks of zoonotic diseases. Further research is needed to clarify the ecological interactions among birds, ticks, and pathogens across different geographic regions.

## Simple Summary

This study investigates the diversity of tick species parasitizing migratory birds in Sardinia and the prevalence of tick-borne pathogens, focusing on *Rickettsia* and *Babesia*/*Theileria* species. Seasonal differences were observed, with *Ixodes ricinus* dominating in autumn and *Hyalomma marginatum* in spring. Molecular analyses revealed a 13.6% prevalence of *Rickettsia* spp., mainly *R. helvetica*, *R. monacensis*, and *R. aeschlimannii*, and a lower prevalence (1.4%) of *Babesia*/*Theileria* species. These findings suggest migratory birds contribute to the dispersal of tick vectors and zoonotic pathogens across geographic regions. These results underscore the importance of integrated surveillance of avian–tick–pathogen interactions to better understand emerging tick-borne disease risks in Mediterranean ecosystems.

## 1. Introduction

In a world facing rapid environmental changes, human activities significantly influence ecosystems, contributing to the decline of numerous animal and plant species. Among the tools used to monitor and understand these changes, bird ringing has proven essential [1]. Researchers can track avian biology, behavior, migratory patterns, productivity, and population dynamics by individually marking birds. Such data are fundamental for conservation and ecological management, especially as species respond to shifting habitats and climate.

Birds, particularly migratory species, play a unique role in ecological networks [1,2]. Many breeding birds in northern Europe undertake seasonal migrations, ranging from short regional movements to intercontinental journeys toward the Southern Hemisphere. These migrations not only allow birds to exploit seasonal resources but also position them as potential vectors of pathogens and parasites, including ticks and tick-borne microorganisms. As migratory birds cross national and ecological boundaries, they may contribute to the dispersal of zoonotic agents across vast geographic areas [3,4].

In addition to acting as vectors of ticks and associated pathogens, migratory birds may also experience direct health consequences from pathogen exposure during migration. It exposes birds to a wide range of environments and novel parasites or pathogens, representing a physiological cost and a potential risk to their survival and fitness [5].

Ticks, as obligate hematophagous ectoparasites, are recognized vectors of a wide variety of zoonotic pathogens. Their distribution and population dynamics are heavily influenced by environmental factors, particularly climate, which affects both tick development and the viability of the pathogens they carry [6]. With global warming, previously unsuitable regions become favorable for tick survival and expansion, increasing the risk of vector-borne disease emergence [7]. In Europe, medically relevant tick species include *Ixodes ricinus*, *Rhipicephalus sanguineus* s.l., *Hyalomma marginatum*, and *Dermacentor reticulatus* [8]. In Sardinia, several of these, including *Rh. sanguineus*, *D. marginatus*, *Haemaphysalis* and *Hyalomma* spp., are commonly found [9,10].

Human exposure to tick-borne pathogens occurs primarily through outdoor activities, agriculture, forestry, and growing urban–wildland interface zones [11]. Moreover, migratory birds can transport ticks over long distances, potentially introducing infected ticks into new areas [12]. These birds may also act as reservoir hosts, especially when the physiological stress of migration compromises their immune systems [13].

Among the emerging tick-borne diseases, rickettsioses caused by members of the Spotted Fever Group *Rickettsia* (SFGR) and protozoan infections, such as babesiosis and theileriosis, have attracted growing attention due to their zoonotic potential and impact on both human and animal health. These pathogens are transmitted by various ixodid ticks, which serve as competent vectors and reservoirs in diverse ecological settings [14].

In Sardinia, multiple studies have highlighted the island’s ecological suitability for the circulation of SFGR, reporting the presence of several *Rickettsia* species, including *R. massiliae* in *R. sanguineus* s.l., *R. aeschlimannii* in *Hyalomma marginatum*, *R. raoultii* in *R. sanguineus* s.l. and *D. marginatus*, and *R*. *slovaca* in *D*. *marginatus* [10,15]. Parallel to these bacterial agents, protozoan parasites such as *Babesia* and *Theileria* spp. have been identified in ticks and blood of both symptomatic and asymptomatic domestic animals, confirming their widespread presence on the island. Molecular investigations have detected *Babesia* and *Theileria* DNA in livestock such as cattle, sheep, pigs, and horses, with notable findings including the first identification of *Theileria annulata* in cattle in Sardinia and a high prevalence of *T. orientalis* in the northern regions. In addition, *B. bigemina* and *T.equi* have been reported in specific host populations and geographic areas [16].

Although the involvement of birds in the ecology of *Borrelia* spp. is well established [17], their contribution to the spread of other pathogens, such as SFGR and piroplasms, is less understood.

Our study addresses this gap by investigating the prevalence and diversity of tick-borne pathogens in ticks collected from migratory birds in Sardinia, focusing on *Rickettsia* and *Babesia*/*Theileria* species. By combining molecular detection methods with ecological surveillance, this research contributes to the broader understanding of vector–pathogen–host interactions. It also supports the development of effective strategies for monitoring and controlling tick-borne zoonoses in Mediterranean ecosystems.

## 2. Materials and Methods

### 2.1. Study Area and Sample Collection

**Figure 1:**
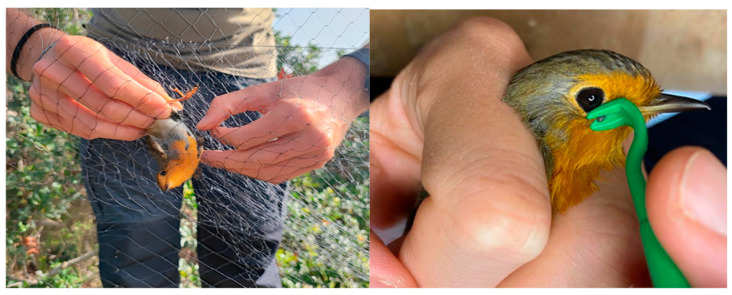
European robin (*Erithacus rubecula*) removal from a mist net during the bird monitoring study and tick removal during field sampling.

This study was carried out in the Asinara National Park, a biodiversity-rich protected area in northwestern Sardinia, strategically positioned along major avian migratory routes in the Mediterranean area. As part of an ornithological ringing program coordinated by ISPRA (Istituto Superiore per la Protezione e la Ricerca Ambientale), birds were captured using mist nets during the spring and autumn migration periods of 2021. Sampling took place at the Tumbarino Ornithological Observatory (one of the most important bird ringing stations in Sardinia) from mid-April to the end of May for spring migration and from mid-September to the end of October for autumn migration. Ticks from birds were gently removed with a sterile tick removal hook (Figure 1), placed in labeled vials, and transported to the Istituto Zooprofilattico Sperimentale della Sardegna (IZS) for laboratory analysis.

### 2.2. Molecular Tick Identification and Pathogen Detection

At the laboratory, ticks were first classified by developmental stage (larva, nymph, and adult) and engorgement status. Species identification was carried out exclusively using molecular methods to ensure high accuracy, particularly for immature stages, which are difficult to distinguish morphologically. Genomic DNA was extracted from individual ticks using the commercial Qiagen DNeasy Blood & Tissue Kit (Qiagen, Hilden, Germany). The mithocondrial 16S rDNA gene was then amplified using PCR using the primers 16S+1 (5′-CTGCTCAATGATTTTTTAAATTGCTGTGG-3′) and 16S-1 (5′-CCGGTCTGAACTCAGATCAAGT-3′), as previously described by Black and Piesnam [18]. The resulting PCR products were sequenced to determine species identity. Molecular identification was also applied to adult ticks to confirm morphological assessments.

Originally collected for a study on *Borrelia* spp. [17], these tick specimens were retrospectively examined to investigate the presence of other tick-borne pathogens, namely, *Rickettsia* spp. and *Babesia/Theileria* spp. Pathogen detection was performed using both real-time and conventional PCR assays targeting the gltA which encodes citrate synthase gene conserved across both pathogenic and non-pathogenic Rickettsia species. Detection of Babesia/Theileria spp. was performed targeting the 18S rRNA gene, as detailed in Table 1.

PCR reaction mixes and thermal cycling conditions were optimized in-house. Real-time PCR was carried out using the Quantifast Probe PCR Kit (Qiagen, Hilden, Germany) in a 20 µL reaction containing 0.5 µM of each primer, 0.25 µM of TaqMan probe, 1.25× Internal Control Assay, and 2 µL of DNA. Cycling conditions consisted of an initial denaturation at 95 °C for 5 min, followed by 50 cycles of 95 °C for 15 s and 60 °C for 30 s. Samples with a Ct values ≤ 45 cycles were considered positive.

Conventional PCRs were performed in 25 µL reaction volumes, each containing 12.5 µL of 6× PCR Master Mix (Qiagen, Hilden, Germany), 1 µM of each primer, 1 µL of DNA template, and nuclease-free water. Cycling conditions included an initial denaturation at 95 °C for 15 min, followed by 40 cycles of denaturation at 95 °C for 30 s, annealing at 60 °C for 30 s (optimized for the 18S rRNA and *gltA* targets) and 72 °C for 1 min, with a final extension at 72 °C for 5 min. Each run included negative (no-template) and positive (DNA extracted from *R. rickettsii* and *B. bovis*) controls.

### 2.3. Purification and Sequencing

All PCR positive amplicons were purified using the QIAquick Spin PCR Purification Kit (Qiagen, Hilden, Germany), following the manufacturer’s protocol. Purified products were subjected to bidirectional Sanger sequencing using the same primers used for amplification. Sequencing was performed on an ABI-PRISM 3500 Genetic Analyzer (Applied Biosystems, Seevetal, Germany) with the dRhodamine Terminator Cycle Sequencing Ready Reaction Kit (Applied Biosystems) in accordance with the manufacturer’s guidelines.

Resulting chromatograms were edited and assembled using ChromasPro v. 1.34 (Technelysium Pty Ltd., Tewantin, Queensland, Australia). Sequences alignment was conducted with CLUSTALX [21] and species-level identification was based on BLASTn analysis against the GenBank database using a ≥99% identity threshold. This approach ensured accurate species determination for both tick vectors and associated pathogens. Pairwise and multiple sequence alignments, as well as identity matrices, were generated using CLUSTALW [21] and BioEdit [22], respectively.

## 3. Results

### 3.1. Tick Species Composition

A total of 961 ticks (862 in autumn and 99 in spring) was collected from 461 migratory birds during the autumn and spring migratory periods of 2021. The autumn sample was dominated by short-distance migrants, primarily local and regional species like the European robin and common blackbird, which accounted for the majority of parasitized individuals (Table 2). Of the 592 larvae collected, 91 (91/592; 15.4%) were fully engorged, 45 (45/592; 7.6%) partially engorged, and 456 (456/592; 77%) non-engorged. Among the 231 nymphs, 64 (64/231; 27.7%) were engorged, 13 (13/231; 5.6%) partially engorged, and 154 (154/231; 66.6%) non-engorged. All adult ticks were fully engorged at the time of collection.

In contrast to autumn, the spring migration was dominated by long-distance migrants like the common redstart and pied flycatcher, which migrate from sub-Saharan Africa to their European breeding grounds (Table 3). During this period, 30 larvae were collected, of which five (5/30; 16.7%) were engorged and 25 unengorged (25/30; 83.3%). Among the 55 adult ticks, 37 (37/55; 67.3%) were engorged and 13 (13/55; 23.6%) unengorged. All 14 nymphs collected in spring were unengorged.

The tick species composition showed notable seasonal variation. In autumn (post-breeding), *Ixodes ricinus* was the predominant species, accounting for 77% of ticks collected. Conversely, *Hyalomma marginatum* predominated in spring (pre-breeding), comprising 75% of the collected ticks. Details of tick–host associations across bird species are shown in Table 2 and Table 3. Despite the application of molecular methods, some immature ticks could only be identified to the genus level (*Ixodes* spp. and *Hyalomma* spp.) due to ambiguous sequence data.

### 3.2. Rickettsia spp. Prevalence During Migration Seasons

Real-time PCR revealed the presence of *Rickettsia* spp. in 13.6% (131/961) of the tested ticks. Among them, 87 were not engorged, 35 were fully engorged, and 8 were partially engorged. Species-level identification based on *gltA* gene sequencing revealed the presence of only pathogenic species in the analyzed samples. *Rickettsia helvetica* was the most frequently detected species (50/131; 38.2%), followed by *Rickettsia monacensis* (49/131; 37.4%) and *Rickettsia aeschlimannii* (29/131; 22.1%). Less frequently detected species included *R. sibirica* (2/131; 1.5%) and *R. raoultii* (1/131; 0.8%).

*R. helvetica* and *R. monacensis* were primarily associated with European robins (*Erithacus rubecula*), blackbirds (*Turdus merula*), and song thrushes (*Turdus philomelos*), and were mostly detected in larvae and nymphs of *I. ricinus*, especially those collected from robins. These two species were also found in *I. frontalis* and *Hy. marginatum*.

*Rickettsia aeschlimannii* was most commonly found in ticks from the woodchat shrike (*Lanius senator*) and was also present in ticks from the common redstart (*Phoenicurus phoenicurus*) and pied flycatcher (*Ficedula hypoleuca*). It was identified in *I. ricinus*, *I. frontalis*, and *Hyalomma* spp., with a strong association observed between *R. aeschlimannii* and *Hyalomma* ticks collected from redstarts and flycatchers. *Ixodes ricinus* was the most frequently detected tick species and was responsible for the majority of *R. helvetica* and *R. monacensis* detections. Larvae represented the most common developmental stage (*n* = 80), particularly in ticks collected from robins, blackbirds, and song thrushes. Nymphs were moderately represented (*n* = 28), especially in *I. ricinus* and *I. frontalis* from blackbirds, song thrushes, and woodchat shrikes. Adult ticks (*n* = 23) were identified primarily in *Hyalomma marginatum* and *Ixodes frontalis*, particularly on redstarts and shrikes. During the spring migration, the most common *Rickettsia* species identified was *R. aeschlimannii*, with 29 positive samples across various stages. Additionally, *R. sibirica* and *R. raoultii* were detected in small numbers (Table 4).

### 3.3. Babesia/Theileria spp. Prevalence During Migration Seasons

PCR targeting piroplasmid DNA revealed 14 positive samples out of 961 ticks analyzed (1.4%). The majority of detections (9/14) were associated with European robins (*Erithacus rubecula*), primarily from *Ixodes ricinus*. Detected pathogens included *B. venatorum* (in two unengorged larvae), *T. ovis* (in four unengorged and one partially engorged larva), *Theileria* sp. *OT3* (in one unengorged larva), *T. orientalis* (from three unengorged and one engorged larva), and *T. equi* (from one unengorged larva). Additionally, one unengorged larva of *Ixodes ventalloi* collected from a robin tested positive for *T. ovis*. Common blackbirds (*Turdus merula*) accounted for four positive samples, involving both larvae and nymphs of *I. ricinus* and *I. frontalis*, and included *B. venatorum*, *B. capreoli*, and *T. orientalis*. One positive larva was also recorded from a song thrush (*Turdus philomelos*), although the genospecies could not be identified. Overall, *T. ovis* (n = 5) and *T. orientalis* (n = 4) were the most frequently detected genospecies, followed by *B. venatorum* (n = 2), *T. equi* (n = 1), *B. capreoli* (n = 1), and *Theileria* sp. *OT3* (n = 1). Most positive ticks were larvae, with a single case detected in a nymph (Table 5).

## 4. Discussion

This study provides a comprehensive insight into the diversity and seasonal dynamics of tick species parasitizing migratory birds, as well as their role in the dissemination of *Rickettsia* and *Babesia*/*Theileria* species. Expanding on the findings of Chisu et al. [17], who originally analyzed these samples for *Borrelia* spp., we retrospectively examined the same tick collection to detect additional tick-borne pathogens. This broader approach contributes to a more integrated understanding of the eco-epidemiology of avian–vector–pathogen interactions.

A clear seasonal difference in tick species composition was observed. *Ixodes ricinus* was the overwhelmingly dominant tick species during the autumn migration, especially among short-distance migratory birds such as the European robin (*Erithacus rubecula*), common blackbird (*Turdus merula*), and song thrush (*Turdus philomelos*). The high proportion of larvae and nymphs in early feeding stages suggests a recent acquisition of ticks during migratory stopovers or local movements within the region. Although bird species were classified according to broad migratory strategy, some short-distance migrants (e.g., *Erithacus rubecula* and *Turdus merula*) are known partial migrants with migratory behavior varying across their range. In Sardinia, particularly during autumn and winter, mixed populations of residents and migrants are likely to co-occur, which may influence tick exposure dynamics (Table 2 and Table 3).

Additionally, while most long-distance migrants in this study were trans-Saharan, *Sylvia atricapilla* represents an exception. Many individuals of this species overwinter within the Mediterranean basin rather than crossing the Sahara. Consequently, these birds likely acquire ticks locally or regionally, indicating that autumn captures largely reflect endemic host–vector dynamics. This is consistent with the findings of Rataud et al. [23] and Dumas et al. [24], who noted that local birds play a central role in sustaining regional tick populations and in maintaining the enzootic transmission of tick-borne pathogens. This interpretation also aligns with the seasonal host–tick associations reported by Chisu et al. [17], who documented a high prevalence of *Borrelia* spp. in immature *Ixodes* ticks collected in autumn from the same bird species.

The increased presence of *Hyalomma marginatum* during spring is of particular epidemiological concern due to their established vector competence for *Rickettsia aeschlimannii* and Crimean-Congo hemorrhagic fever virus, pointing out the potential for avian-mediated introductions of vector-borne pathogens into new ecological settings [25,26]. These data reinforce the critical role of migratory birds in shaping the spatiotemporal distribution of tick species and in facilitating the long-range movement of zoonotic pathogens.

Larvae (representing the 64.7% of the ticks collected) were the most commonly collected tick stage in autumn, followed by nymphs (25.5%) and, to a lesser extent, adults (9.8%). This pattern was seen for *Ixodes ricinus*, a species that has a life cycle synchronized to maximize larval activity in late summer and early autumn [27]. Moreover, the predominance of larvae reflects the typical host-seeking behavior of these life stages, which are adapted to parasitize smaller, ground-foraging vertebrates such as passerine birds [28,29]. Similarly, Dumas et al. [24] and Chisu et al. [17] noted that larval ticks are the primary stage collected from small-to-medium-sized avian hosts, aligning with their role in the early stages of the enzootic transmission cycle. The ground-foraging ecology of many of the birds sampled (such as robins and thrushes) further increases their exposure to larvae and nymphs, which remain close to the substrate during their questing phase. This host–stage association reinforces the importance of avian ecology in shaping tick-host dynamics and has implications for understanding how immature tick stages are distributed across habitats and hosts.

A rickettsial infection prevalence of 13.6% was observed in ticks associated with birds. The most commonly detected species were *R. helvetica*, *R. monacensis*, and *R. aeschlimannii*, reflecting their known associations with ixodid ticks in the Palearctic region [30]. In this study, *R. helvetica* and *R. monacensis* were primarily associated with immature stages of *Ixodes ricinus*, particularly those collected from European robins, blackbirds, and song thrushes. These passerines, due to their ground-foraging behavior and high tick-infestation rates, may serve as both biological amplifiers and ecological dispersers of these pathogens. Notably, the detection of *R. helvetica* in *I. ricinus* from European robins (74.4%) suggests a potential reservoir competence, a hypothesis supported by previous observations of transovarial and transstadial transmission in *I. ricinus* populations [31,32]. Given its association with serious human conditions, including perimyocarditis and neurological syndromes such as Bell’s palsy [33,34], the widespread distribution of *R. helvetica* is of increasing public health interest.

Similarly, *R. monacensis*, a recognized human pathogen, was also frequently detected in *I. ricinus* and *I. frontalis* ticks collected from robins and blackbirds [35]. While *I. ricinus* remains the principal vector, the presence of *R. monacensis* in ticks feeding on multiple bird species suggests that blackbirds, and potentially robins, may play a reservoir role. The identification of this pathogen in both larvae and nymps supports the potential for vertical transmission and the persistence across tick generations.

*Rickettsia aeschlimannii* was more commonly associated with adult *Hyalomma* ticks [36], particularly *Hy. marginatum* and *Hy. rufipes*, and, to a lesser extent, adult *I. ricinus* and *I. frontalis*. These ticks were largely collected from long-distance migratory birds, such as those in Table 4, suggesting the introduction of *R. aeschlimannii* from North African or Mediterranean regions during spring migration. Given its pathogenicity in humans and its wide tick host range, this species warrants careful surveillance, particularly in migratory bird stopover sites and breeding areas.

The occasional detection of *R. sibirica* and *R. raoultii*, both medically relevant but less commonly reported in Western Europe, further indicates the broad biogeographical range of ticks transported by birds. *R. sibirica* was detected in *I. ricinus* ticks from redstarts, a species that migrates between Europe and Africa, suggesting its possible involvement in transcontinental pathogen dispersal. These detections emphasize the role of migratory birds as vehicles for the introduction of exotic or emerging *Rickettsia* species into non-endemic regions.

Seasonal variation in tick species composition was also evident, with *I. ricinus* predominating in the autumn and *Hyalomma marginatum* being more common in spring. These dynamics can influence which *Rickettsia* species are introduced or amplified at specific times of year, potentially shaping local disease risk. Although the number of ticks collected during spring migration was markedly lower than in autumn, this likely reflects the reduced sample size of birds examined in spring rather than a true seasonal decline in tick prevalence. Despite this limitation, a high infestation rate was recorded, particularly among long-distance migrants such as the common redstart and pied flycatcher. These findings suggest that sampling effort, rather than ecological factors alone, may partially explain the seasonal differences in tick abundance.

Piroplasmid DNA was detected in 1.4% of examined ticks (14/961), indicating a low but ecologically relevant prevalence of *Babesia* and *Theileria* spp. in avian-associated tick populations. The ticks were primarily *Ixodes ricinus* larvae collected from European robins and blackbirds. Notably, most of these were not engorged, which suggests that pathogens may not result from recent bloodmeal, raising the possibility of alternative transmission routes, such as transovarial or environmental acquisition. While vertical transmission has been reported for some *Babesia* spp., it remains unconfirmed for *Theileria* spp., raising questions about the origin and viability of these infections in immature tick stages.

Among the identified pathogens, *B. venatorum* emerged as a noteworthy zoonotic agent. Two *I. ricinus* larvae from a robin and a blackbird tested positive for this species, which was first reported in migratory birds in Norway and is now recognized as a pathogen capable of infecting humans [37]. *B. venatorum* is primarily associated with roe deer (*Capreolus capreolus*), and adult *I. ricinus* ticks commonly parasitize these mammals [38]. Its detection in immature ticks supports the possibility of transovarial transmission, facilitating the maintenance of the pathogen even in the absence of its principal vertebrate host. Further investigation is needed to determine whether birds carry *Babesia* spp. in their bloodstream, and it could clarify their potential role as biological or mechanical vectors.

Similarly, *B. capreoli*, another species associated with roe deer, was detected in a single *I. ricinus* nymph. Like *B. venatorum*, it is suggested to be transmitted transovarially within ticks, as inferred from its occasional detection in larval stages [38].

The presence of *Theileria* spp., including *T. orientalis*, *T. ovis*, and *T. equi*, further supports the circulation of apicomplexan parasites on the island and their potential dissemination through birds [16,39]. Although birds are not competent reservoirs for these pathogens, they may act as mechanical carriers of infected ticks, thereby contributing to the ecological spread of these pathogens. Notably, *T. ovis* was detected in *I. ventalloi*, a tick species not traditionally associated with livestock [40]. This finding raises important questions about overlooked vectors in the epidemiology of ovine theileriosis and highlights the need to investigate the vector competence across a broader range of tick species.

Importantly, the molecular approach employed in this study did not confirm whether the detected pathogens were viable or being transmitted. The absence of data on parasite developmental stages or the origin of the bloodmeal origin limits definitive conclusions about the dynamics of pathogen transmission. Nevertheless, the detection of piroplasmid DNA in unengorged larvae, especially for *Theileria* spp., for which transovarial transmission has not been established, prompts further investigation. Further experimental studies are needed to explore the dynamics of parasite viability, transmission potential, and host–vector competence. Given the limited number of positive samples, particularly the single detection of piroplasmids in a nymph, further studies with larger sample sizes are necessary to confirm the role of migratory birds and their ticks in the ecology of *Babesia* and *Theileria* spp.

The concurrent detection of multiple *Rickettsia*, *Babesia*, and *Theileria* species in bird-associated ticks highlights the importance of integrated eco-epidemiological surveillance at the interface of wildlife, vectors, and zoonotic pathogens. Although our findings suggest that birds may play a role in the ecology of these pathogens through their association with infected ticks, we did not directly analyze avian blood samples to confirm their status as competent reservoirs. Therefore, birds in this study can be considered only potential carriers rather than confirmed reservoirs. Future studies involving direct sampling of birds will be necessary to clarify their reservoir competence.

## 5. Conclusions

This study highlights the relationship between migratory birds, tick vectors, and the dissemination of various zoonotic pathogens such as *Rickettsia*, *Babesia*, and *Theileria* species. These findings not only reinforce the role of birds in the ecological dynamics of tick-borne diseases, but also emphasize the need for integrative, One Health surveillance approaches that consider the interconnectedness of wildlife, vectors, and human health. With shifting climate patterns, habitat alterations, and changing migratory behaviors, continued monitoring of tick populations and pathogen prevalence is essential to understand and mitigate the risks of pathogen introduction and spread. As such, ongoing research into the role of migratory birds in tick-borne pathogen dynamics is critical to anticipating emerging public health and veterinary challenges.

## Figures and Tables

**Table 1:** Primers sets used for the detection of *Rickettsia* spp. and piroplasmida DNA.

Pathogen	Target Gene	Primer/Probe	Sequence	References
***Rickettsia *** **spp.**	***gltA***	**CS5 Forw**	s5′-GAGAGAAAATTATATATCCAAATGTTGAT-3′	[19]
		**CS5 Rev**	5′-AGGGTCTTCGTGCATTTCTT-3′	
		**CS5 Probe**	FAM-CATTGTGCCATCCAGCCTACGGT -BHQ1	
***Rickettsia *** **spp.**	***gltA***	***gltA *** **For**	5′-CCTATGGCTATTATGCTTGC-3′	[10]
		***gltA *** **Rev**	5′-ATTGCAAAAAGTACAGTGAACA -3′	
**Piroplasmida**	**5.8S rRNA gene**	**5.8S Forw**	5′-AYYKTYAGCGRTGGATGTC-3′	[20]
		**5.8S Rev**	5′-TCGCAGRAGTCTKCAAGTC-3′	
		**5.8S Probe**	FAM-TTYGCTGCGTCCTTCATCGTTGT-MGB	
**Piroplasmida**	**18S rRNA gene**	**BJ1 For**	5′-GTCTTGTAATTGGAATGATGG-3′	[16]
		**BN2 Rev**	5′-TAGTTTATGGTTAGGACTACG-3′	

**Table 2:** Tick infestation data collected from parasitized migratory birds during autumn migration, including tick developmental stages (larvae (L), nymphs (N), and adults (A)), migratory behavior classification of each bird species, and the percentage of individuals parasitized per species. Some data are adapted from Chisu et al. [17].

Season	Bird Species *(Scientific name)*	Migration Type	Tested Ticks (n% of Total Ticks)	Parasitized Birds (n% of Total Birds)	Birds Parasitized/Examined	Tick Species Associated	L	N	A	Total
**Autumn**	European robin (*Erithacus rubecula*)	Short-distance	579 (67%)	301 (74.3%)	301/579 (52%)	*I. ricinus*	284	100	14	**398**
						*I. frontalis*	78	24	8	**110**
						*I. inopinatus*	1	0	0	**1**
						*I. ventalloi*	4	0	0	**4**
						*Ixodes* spp.	39	24	3	**66**
	Common blackbird (*Turdus merula*)	Short-distance	188 (21.8%)	54 (13.3%)	54/188 (28.7%)	*I. frontalis*	1	1	1	**3**
						*I. ricinus*	128	56	0	**184**
						*I. ventalloi*	0	1	0	**1**
	Song thrush (*Turdus philomelos*)	Short-distance	68 (8%)	34 (8.4%)	34/68 (50%)	*I.ricinus*	43	22	1	**66**
						*I. inopinatus*	2	0	0	**2**
	Woodlark (*Lullula arborea*)	Short-distance	17 (2%)	7 (1.7%)	7/17 (41.2%)	*I. ricinus*	1	0	11	**12**
						*Hy. marginatum*	2	2	1	**5**
	Eurasian blackcap (*Sylvia atricapilla*)	Long-distance	4 (0.5%)	4 (1%)	4/4 (100%)	*I. frontalis*	4	0	0	**4**
	Garden warbler (*Sylvia borin*)	Long-distance	2 (0.2%)	2 (0.5%)	2/2 (100%)	*I. ricinus*	1	1	0	**2**
	Common redstart (*Phoenicurus phoenicurus*)	Long-distance	3 (0.4%)	2 (0.5%)	2/3 (66.7%)	*I. ricinus*	3	0	0	**3**
	Little owl (*Athene noctua*)	Resident	1 (0.1%)	1 (0.3%)	1/1 (100%)	*I. ricinus*	1	0	0	**1**
**TOTAL**	-	-	**862**	**405**	-	-	**592**	**231**	**39**	**862**

Notes: (1) Short-distance migrants include partial migrants, with mixed populations of residents and seasonal migrants in Sardinia. (2) Long-distance migrants include trans-Saharan migrants. (3) Resident species are non-migratory.

**Table 3:** Tick infestation in migratory and resident birds during spring season: prevalence, tick species, and developmental stages. Data partly adapted from Chisu et al. [17].

Season	Bird Species *(Scientific name)*	Migration Type	Tested Ticks (n% of Total Ticks)	Parasitized Birds (n% of Total Birds)	Birds Parasitized/Examined	Tick Species Associated	L	N	A	Total
**Spring**	Common redstart (*Phoenicurus phoenicurus*)	Long-distance	56 (57%)	25 (44.6%)	25/56 (44.6%)	*Hy. marginatum*	7	5	23	**35**
						*Hy. rufipes*	2	1	14	**17**
						*Rh. bursa*	1	0	0	1
						*I. inopinatus*	1	0	0	1
						*I. ventalloi*	0	1	0	1
						*I. frontalis*	1	0	0	1
	Wood warbler (*Phylloscopus sibilatrix*)	Long-distance	11 (11%)	7 (12.5%)	7/11 (63.6%)	*Hy. marginatum*	5	0	6	**11**
	European pied flycatcher (*Ficedula hypoleuca*)	Long-distance	8 (8%)	7 (12.5%)	7/8 (87.5%)	*Hy. marginatum*	1	4	3	**8**
	European robin (*Erithacus rubecula*)	Short-distance	10 (10%)	5 (8.9%)	5/10 (50%)	*Hy. marginatum*	9	0	1	**10**
	Common whitethroat (*Sylvia communis*)	Long-distance	3 (3%)	3 (5.3%)	3/3 (100%)	*Hy. marginatum*	0	0	3	**3**
	Willow warbler (*Phylloscopus trochilus*)	Long-distance	2 (2%)	2 (3.6%)	2/2 (100%)	*Hy. marginatum*	0	0	2	**2**
	Common nightingale (*Luscinia megarhynchos*)	Long-distance	3 (3%)	2 (3.6%)	2/3 (66.6%)	*Amblyomma marmoreum*	0	0	1	**1**
						*Hy. marginatum*	2	0	0	**2**
	Song thrush (*Turdus philomelos*)	Short-distance	2 (2%)	1 (1.8%)	1/2 (50%)	*Hy. marginatum*	1	1	0	**2**
	Whinchat (*Saxicola rubetra*)	Long-distance	1 (1%)	1 (1.8%)	1/1 (100%)	*Hyalomma* spp.	0	1	0	**1**
	Tyrrhenian spotted flycatcher (*Muscicapa striata tyrrhenica*)	Long-distance	1 (1%)	1 (1.8%)	1/1 (100%)	*I. frontalis*	0	1	0	**1**
	Eurasian magpie (*Pica pica*)	Resident	1 (1%)	1 (1.8%)	1/1 (100%)	*Hy. marginatum*	0	0	1	**1**
	Woodchat shrike (*Lanius senator*)	Long-distance	1 (1%)	1 (1.8%)	1/1 (100%)	*I. frontalis*	0	0	1	**1**
**TOTAL**	-	-	**99**	**56**	-	-	**30**	**14**	**55**	**99**

Notes: (a) L = larvae; N = nymphs; A = adults. (b) Percentages refer to the proportion relative to the total number of ticks or birds per species during the spring season. Absolute numbers are reported outside parentheses; percentages are shown inside parentheses. (c) Migration types: Short-distance migrants include partial migrants; long-distance migrants include trans-Saharan species; resident species are non-migratory.

**Table 4:** Distribution of *R. helvetica*, *R. monacensis*, *R. aeschlimannii*, *R. sibirica*, and *R. raoultii* according to bird host species, tick species, and tick developmental stage (Larva [L], Nymph [N], Adult [A]).

Bird Species	Tick Species	Tick Stage	*R. helvetica*	*R. monacensis*	*R. aeschlimannii*	*R. sibirica*	*R. raoultii*	Total
European robin (*Erithacus rubecula*)	*I. ricinus*	L	31	26	0	0	0	57
	*I. ricinus*	N	5	4	0	0	0	9
	*I. frontalis*	L	0	3	0	0	0	3
Blackbird (*Turdus merula*)	*I. ricinus*	L	6	3	0	0	0	9
	*I. ricinus*	N	4	5	0	0	0	9
	*I. frontalis*	L	0	1	0	0	0	1
	*I. frontalis*	N	1	0	0	0	0	1
	*Hy. marginatum*	N	0	1	0	0	0	1
Song thrush (*Turdus philomelos*)	*I. ricinus*	L	2	3	0	0	0	5
	*I. ricinus*	N	0	2	0	0	0	2
	*I. frontalis*	L	0	1	0	0	0	1
Woodchat shrike (*Lanius senator*)	*I. ricinus*	A	0	0	3	0	0	3
	*I. ricinus*	N	0	0	1	0	0	1
	*I. frontalis*	A	0	0	2	0	0	2
	*I. frontalis*	N	0	0	2	0	0	2
	*Hy. marginatum*	L	0	0	1	0	0	1
Common redstart (*Phoenicurus phoenicurus*)	*I.ricinus*	A	1	0	0	0	0	1
	*I. ricinus*	A	0	0	9	2	0	11
	*I. ricinus*	N	0	0	2	0	0	2
	*I. ricinus*	L	0	0	2	0	0	2
	*Hy. marginatum*	N	0	0	1	0	0	1
	*Hy. rufipes*	A	0	0	3	0	0	3
Pied flycatcher (*Ficedula hypoleuca*)	*I. ricinus*	A	0	0	2	0	0	2
Whitethroat (*Sylvia communis*)	*I. ricinus*	A	0	0	1	0	0	1
Nightingale (*Luscinia megarhynchos*)	*I. ricinus*	L	0	0	0	0	1	1
**Total**			**50**	**49**	**29**	**2**	**1**	**131**

**Table 5:** Distribution of piroplasmids by tick species and life stage on selected passerine hosts.

Bird Species	Tick Species	Tick Stage	*B. venatorum*	*B. capreoli*	*T. ovis*	*T.* sp. *OT3*	*T. orientalis*	*T. equi*	Total
European robin (*Erithacus rubecula*)	*I. ricinus*	L	1	0	2	1	1	1	**6**
	*I. frontalis*	L	0	0	0	0	1	0	1
	*I. ventalloi*	L	0	0	1	0	0	0	1
	*I. ricinus*	L	0	0	1	0	0	0	1
Common blackbird (*Turdus merula*)	*I. ricinus*	L	1	0	0	0	0	0	1
	*I. ricinus*	N	0	1	0	0	0	0	1
	*I. ricinus*	L	0	0	1	0	0	0	1
	*I. frontalis*	L	0	0	0	0	1	0	1
Song thrush (*Turdus philomelos*)	*I. ricinus*	L	0	0	0	0	1	0	1
**TOTAL**			**2**	**1**	**5**	**1**	**4**	**1**	**14**

## Data Availability

The data collected during this study are available upon request from the corresponding author.
